# Reply to Fernández-Flores, Á. Desmoplakin-Related Arrhythmogenic Cardiomyopathy Carriers and Desmosomal-Type Acantholysis: A Previous Description of the Keratinocyte “Fingerprint Sign”. Comment on “Metze et al. Desmosomal-Type Acantholysis—A New Histologic Pattern Related to Mutations of Genes for Desmosomal Proteins. *Dermatopathology* 2026, *13*, 17”

**DOI:** 10.3390/dermatopathology13030040

**Published:** 2026-09-02

**Authors:** Dieter Metze, Kira Süßmuth

**Affiliations:** 1 Dermatopathology Unit, Department of Dermatology, University Hospital Münster, 48129 Muenster, Germany; 2 Helios Klinikum Berlin-Buch, Schwanebecker Chaussee 50, 13125 Berlin, Germany

We are grateful for the commentary by Ángel Fernández-Flores on our concept of “desmosomal-type acantholysis”, a distinct histologic form of acantholysis associated with mutations in genes encoding desmosomal proteins [1]. We defined this characteristic histologic pattern by distinct widening of the intercellular spaces accompanied by hypereosinophilic keratinocytes with regular nuclei, a constellation that differs from conventional forms of acantholysis. Furthermore, we demonstrated that “desmosomal-type acantholysis” may occur not only in sporadic solitary acanthoma, but also as an incidental finding in melanocytic nevi [2].

The histological spectrum of “desmosomal-type acantholysis” appears to be broad, as mutations in genes encoding desmosomal proteins are associated with diverse ultrastructural abnormalities, including reduced or malformed desmosomes and corneodesmosomes, loss or displacement of the inner plaque with impaired attachment of keratin filaments, clumping or dissolution of the keratin cytoskeleton, and widening of the intercellular spaces up to complete acantholysis [3].

In addition to the isolated widening of intercellular spaces within the stratum spinosum observed in striate palmoplantar keratosis, widening of the intercellular spaces may also be accompanied by psoriasiform dermatitis, as reported in severe dermatitis–multiple allergies–metabolic wasting (SAM) syndrome [4]. Whereas these alterations are associated with mutations affecting desmoglein or desmoplakin, other morphologic patterns appear to be more specific to individual desmosomal protein defects. Mutations involving plakophilin-coding genes in ectodermal dysplasia–skin fragility syndrome result in intracytoplasmic hypereosinophilic globules within suprabasal keratinocytes [5], whereas inflammatory peeling skin syndrome is characterized by loss of corneodesmosomes associated with intercellular splitting of corneocytes originating from the stratum granulosum [6].

Ángel Fernández-Flores, Cabrera-Borrego, and co-authors have recently described an additional histological variant of “desmosomal-type acantholysis” in clinically unaffected skin from a cohort of heterozygous carriers of truncating desmoplakin (DSP) variants associated with arrhythmogenic cardiomyopathy [7]. The authors identified a distinctive “fingerprint-like pattern” within the cytoplasm of suprabasal keratinocytes, most likely reflecting a characteristic filamentous reorganization of the keratin cytoskeleton in desmoplakin deficiency. Particularly noteworthy was the focal occurrence of intraepidermal clefting and roundish, fully acantholytic cells, an unusual feature in “desmosomal-type acantholysis”, in which widening of the intercellular spaces is generally the predominant histologic finding.

Taken together, these observations further emphasize the diagnostic value of skin biopsies in patients with suspected mutations in genes encoding desmosomal proteins, even in the absence of overt cutaneous manifestations. Further studies are warranted to clarify potential correlations between specific histological patterns and the underlying genetic defects.

## Data Availability

No new data were created or analyzed in this study. Data sharing is not applicable to this article.
